# Case Report: Tocilizumab for the Treatment of SARS-CoV-2 Infection in a Patient With Aplastic Anemia

**DOI:** 10.3389/fonc.2020.562625

**Published:** 2020-09-18

**Authors:** Gina Keiffer, Zachary French, Lindsay Wilde, Joanne Filicko-O'Hara, Usama Gergis, Adam F. Binder

**Affiliations:** ^1^Sidney Kimmel Cancer Center, Thomas Jefferson University, Philadelphia, PA, United States; ^2^Department of Medicine, Thomas Jefferson University Hospital, Philadelphia, PA, United States

**Keywords:** COVID-19, SARS-CoV-2, tocilizumab, cytokine storm, aplastic anemia

## Abstract

While cytokine storm develops in a minority of patients with severe acute respiratory syndrome coronavirus 2 (SARS-CoV-2) infection, novel treatment approaches are desperately needed for those in whom it does. Tocilizumab, an interleukin-6 receptor antibody, has been utilized for the treatment of cytokine storm in a number of severe inflammatory conditions, including in patients with severe coronavirus disease 2019 (COVID-19). Here, we present the first published case utilizing this therapy in a patient with underlying immunodeficiency. Our patient with aplastic anemia developed cytokine storm due to COVID-19 manifested by fever, severe hypoxia, pulmonary infiltrates, and elevated inflammatory markers. Following treatment with tocilizumab, cytokine storm resolved, and the patient was ultimately safely discharged from the hospital.

## Introduction

To date, the novel SARS-CoV-2 has infected more than 4,500,000 people in the United States and more than three times that number worldwide. While a minority of patients develop severe symptoms from COVID-19 requiring hospitalization, treatment options for those who do are limited. There is no currently accepted standard and new approaches are desperately needed.

Following the initial reports of COVID-19 from Wuhan, China, Huang et al. (1) described the clinical features of the disease. The subset of patients who required intensive care was found to have significant increase in inflammatory cytokines (1). Others have since shown that an increase in interleukin (IL)-6 is associated with critical illness (2) and mortality (3) in COVID-19 patients.

Treatment with tocilizumab, an anti-IL-6 receptor (IL-6R) antibody, has been proposed as a strategy to control cytokine storm associated with COVID-19 (4, 5). This strategy has been utilized by others in the treatment of cytokine storm associated with SARS-CoV-2 (6–9). We describe here the first reported use of tocilizumab in a patient with underlying immune dysregulation, specifically aplastic anemia (AA). In our patient with underlying immunodeficiency, tocilizumab successfully reversed cytokine storm due to COVID-19 and the patient was successfully discharged from the hospital.

## Case Description

A 72-year-old man with a past medical history of AA, amegakaryocytic thrombocytopenia and chronic kidney disease (CKD) stage 3 presented in April 2020 with high fever, cough, and progressive fatigue.

Aplastic anemia had been diagnosed in 2014 and initially responded to therapy with horse anti-thymocyte globulin (ATG), methylprednisolone and cyclosporine A. Nine months later, his disease progressed and he was treated with rabbit ATG, methylprednisolone, and cyclosporine A. In late 2019, his disease progressed primarily as thrombocytopenia due to acquired amegakaryocytic thrombocytopenia (AAT). He received four doses of rituximab in early 2020 for treatment of AAT without response. Bone marrow biopsy performed 2 months prior to presentation was hypocellular with 15–20% bilineage hematopoiesis and near absence of megakaryocytes without evidence of dysplasia or increased blasts. While his diagnosis is most accurately classified as AAT at the time of presentation, he had been consistently pancytopenic for 4 months despite treatment with cyclosporine A and remained dependent on platelet transfusions. Family and psycho-social history were non-contributory.

**Figure 1:**
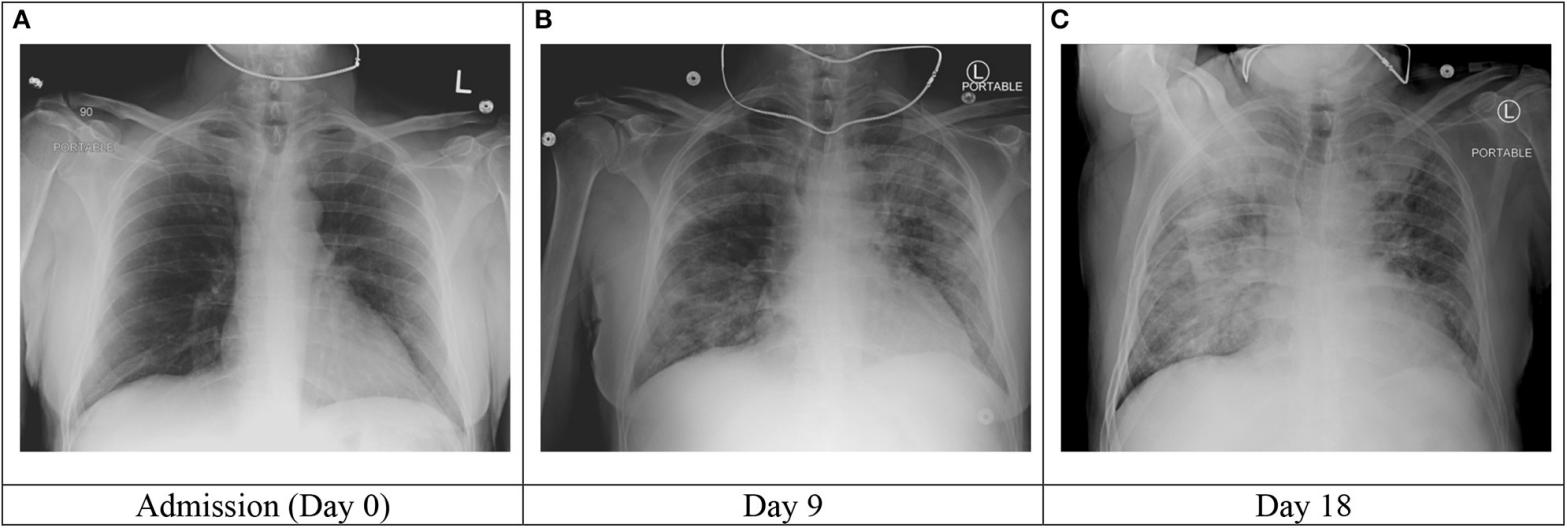
Select chest radiograph images during the patient's hospitalization.

Physical examination at the time of presentation was notable for fever of 103.7F and mild tachycardia (HR 104) with normal oxygen saturation (97%) without supplemental oxygen. He appeared in mild respiratory distress, but pulmonary examination was unremarkable. His initial laboratory studies were notable for severe lymphopenia, which was different from his baseline pancytopenia and elevated C-reactive protein (CRP) and IL-6 as noted in Table 1. Chest radiograph revealed a consolidation in the left midlung, possibly representative of pneumonia (Figure 1A).

**Table 1:** Notable laboratory studies at baseline and throughout hospitalization.

	**Normal range**	**Baseline Day−30**	**Admission Day 0**	**Day 5**	**Day 9**	**Tocilizumab Day 11**	**Day 12**	**Day 14**	**Day 21**	**Day 28**	**Discharge Day 31**
WBC	4.0–11.0 × 10^9^ cells/L	2.5	2.6	0.4	0.7	0.5	0.3	0.4	0.1	0.5	0.5
ANC	1.6–8.0 × 10^9^ cells/L	1.5	2.36	3.00	5.00	0.460	0.220	0.360	–	0.210	0.220
ALC	0.8–4.8 × 10^9^ cells/L	0.800	0.080	0.070	0.130	0.030	0.050	0.030	–	0.220	0.170
Hb (g/dL)	14.0–17.0 g/dL	9.2	8.1	6.1	8.2	6.8	8.0	8.3	7.4	7.0	6.6
Platelet	140–400 × 10^9^ cells/L	24	10	20	24	9	14	10	19	24	18
Creatinine	0.7–1.4 mg/dL	2.34	2.59	2.1	2.34	3.04	2.7	1.91	2.00	1.31	1.6
D-dimer	<230 ng/mL	NR	673	1,005	1,781	1,833	2,209	997	1,932	1,010	960
PT	9.4–13.0 s	–	12.4	–	16.5	–	–	–	16.1	17.0	13.5
aPTT	25–36 s	–	29	–	32	–	–	–	28	28	29
LDH	125–240 IU/L	267	206	232	296	–	272	209	180	142	122
Ferritin	30–400 ng/mL	NR	–	4,122	7,535	7,291	11,363 (Day 13)	6,339	2,541	2,326	2,533
CRP	<0.80 mg/dL	NR	5.10	18.70	34.70	40.10	32.60	8.20	7.20	2.80	1.4
IL-6	<5.00 pg/mL	NR	58.96	–	–	–	–	–	–	1223.8	–
SARS-CoV-2 PCR	Negative	NR	Positive					Positive	Negative (Day 17)	Positive (Day 29)	

*WBC, white blood cell count; ANC, absolute neutrophil count; ALC, absolute lymphocyte count; Hb, hemoglobin; PT, prothrombin time; aPTT, activated partial thromboplastin time; LDH, lactate dehydrogenase; CRP, C-reactive protein; NR, not reported*.

The patient was treated empirically with hydroxychloroquine (400 mg twice a day for 1 day, followed by 200 mg twice a day for 4 days) for possible COVID-19 in the setting of immunosuppression. Ceftriaxone and azithromycin were added for possible community acquired pneumonia. On admission day 0, SARS-CoV-2 PCR nasal swab came back positive. For the first several days of the hospitalization, the patient had persistent fever (range 100–104F) and intermittently required oxygen up to 2 L/min via nasal cannula to maintain oxygen saturation above 94%.

By hospital day 5, he had developed progressive pancytopenia and required near daily transfusion of packed red blood cells and platelets. Due to persistent high fever, and now neutropenia, antibacterial coverage was broadened to cefepime, then meropenem, and antifungal prophylaxis with posaconazole was added. He was also started on tbo-filgrastim and eltrombopag for management of severe neutropenia and thrombocytopenia.

On hospital day 9, the patient developed lethargy and hypoxia requiring supplemental oxygen of 6 L/min via nasal cannula and subsequently Venturi mask at 50% FiO2. Repeat chest radiograph demonstrated significant worsening with bilateral pulmonary infiltrates (Figure 1B). Inflammatory markers, including ferritin and CRP were significantly increased and acute on chronic renal failure worsened (Table 1).

The clinical picture was consistent with cytokine storm due to severe COVID-19 and the patient promptly received tocilizumab 5 mg/kg (400 mg) on hospital day 11. The patient received a single dose of methylprednisolone 60 mg IV immediately prior to tocilizumab administration but did not otherwise receive corticosteroid treatment during his admission. The same day, the patient's fever resolved and he developed mild hypothermia (minimum 94.6F). Over the next several days, his temperature normalized, oxygen requirements steadily declined, inflammatory markers decreased and acute renal failure resolved. The patient remained pancytopenic and transfusion dependent. Meropenem was discontinued and he was transitioned to oral levofloxacin prophylaxis due to persistent neutropenia.

On hospital day 18, the patient developed mild recurrent fever (100.5F, repeat 100.9F). The following day, his fever increased significantly to 103.9F and his oxygen requirement increased to 10 L/min. Chest radiograph revealed new pulmonary infiltrates (Figure 1C). Cefepime was resumed and vancomycin and therapeutic voriconazole were added. Despite these efforts, the patient developed mild hypotension (BP 90/40) which resolved with fluid resuscitation. Antibiotics were further broadened to meropenem and the patient received a single dose of tobramycin. Inflammatory markers at this time were relatively stable.

By hospital day 20, the patient had again clinically improved. His blood pressure remained within normal range and his oxygen requirement decreased to 6 L/min by nasal cannula. The exact cause of his brief clinical deterioration was unclear but was presumed to be due to a secondary bacterial pneumonia. Over the subsequent days, he completed a 7-day course of meropenem and vancomycin. He remained afebrile on this treatment and his oxygen requirement slowly normalized. His repeat IL-6 level on day 28 was significantly elevated at 1223.8 pg/mL.

The patient was discharged home on hospital day 31. He remained pancytopenic and transfusion dependent at the time of hospital discharge. Notably, his SARS-CoV-2 PCR testing has remained persistently positive as of hospital day 29 (with the exception of a single negative result on day 17, which was thought to be a false negative). It is unclear whether detectable viral RNA represents persistent infectiousness or whether non-infectious RNA fragments are being detected. Twenty two days after hospital discharge, SARS-CoV-2 PCR was repeated and was negative.

## Discussion

Hyperinflammatory syndromes, such as the cytokine storm observed in patients with severe SARS-CoV-2 infection, play a major role in the development of critical illness associated with viral infections. Multiple reports have identified significant increases in inflammatory markers, including IL-6, in patients with severe SARS-CoV-2 infection (1, 3, 10–12). In one retrospective analysis, elevated ferritin (mean 1297.6 vs. 614.0 ng/mL), CRP (mean 34.1 vs. 126.6 mg/L), and IL-6 levels (mean 6.8 vs. 11.4 pg/mL) have been identified as risk factors for mortality (3). Significant elevation in IL-6 has previously been identified as a significant factor in the development of cytokine release syndrome (CRS) in patients undergoing chimeric antigen receptor T cell (CAR-T) therapy, as well (13).

IL-6 serves many important immunologic and hematopoietic functions, including differentiation of activated B cells to immunoglobulin-producing plasma cells, differentiation of naïve CD4+ T cells to Th17 cells and of CD8+ T cells to cytotoxic T cells, promotion of T follicular helper cell differentiation, inhibition of regulatory T cell (Treg) differentiation and stimulation of acute-phase proteins (14). In combination with erythropoietin and IL-3, IL-6 promotes formation of myeloid, erythroid, megakaryocyte, and macrophage colonies (15). Tocilizumab is a recombinant, humanized monoclonal antibody which binds and inhibits the soluble IL-6 receptor and has been utilized in the treatment of auto-inflammatory conditions (16) and CRS following CAR-T therapy (17). Based on the success of treating CAR-T-associated CRS with anti-IL-6 therapy, the same strategy has been proposed for the treatment of cytokine storm associated with severe SARS-CoV-2 infection (4, 5). Many case reports and case series support the use of tocilizumab in this setting (6–9) and multiple clinical trials investigating this treatment are underway in the United States and abroad (NCT04317092, NCT04331795, NCT04335071, NCT04356937, and others).

The most promising data thus far are from a retrospective analysis of the use of 1–2 doses of tocilizumab in 21 adult patients with severe or critical COVID-19 treated at two centers in China. Following treatment, all patients became afebrile and 70% had decreased oxygen requirements. Nineteen patients (90.5%) had been discharged from the hospital, including two critical patients, after a mean of 13.5 days following treatment with tocilizumab. No patients developed recurrent fever or symptoms of recurrent pneumonia (9).

A retrospective analysis of 30 French patients age <80 years with severe COVID-19 treated with tocilizumab after at least 5 prior days of illness requiring at least 6 L/min of oxygen therapy who developed rapidly deteriorating COVID-19 pneumonia (increase by more than 3 L/min oxygen therapy in 12 h) showed that treatment with tocilizumab 8 mg/kg for 1–2 doses was associated with decreased ICU admission (OR 0.17, 95% CI 0.06–0.48, *p* = 0.001) and mechanical ventilation (OR 0.42, 95% CI 0.20–0.89, *p* = 0.025) compared to matched controls. No benefit in mortality was seen after weighted analysis (18).

The clinical response observed in our AA patient following treatment with tocilizumab is in line with previously published reports despite his underlying immunodeficiency. After a single dose of tocilizumab, his clinical and laboratory markers of severe COVID-19 had improved. While he did experience a brief clinical deterioration 7–8 days following tocilizumab administration, this was thought to be due to secondary bacterial pneumonia as evidenced by the pattern change in the chest radiologic image, and unlikely related to use of tocilizumab.

Both the development of cytokine storm and the resolution thereof following tocilizumab is particularly notable in our patient in light of his underlying immune dysfunction. Activation of aberrant T cells and monocytes, which produce large numbers of inflammatory cytokines, appears to be a driving factor and has been implicated in many patients with severe COVID-19 (9, 11, 19). Guo et al. (20) profiled the peripheral immune cells of two severe SARS-CoV-2-infected patients at three time points (severe stage: 12 h, recovery stage: 5 days and healthy stage: 7 days) following treatment with tocilizumab to better understand the immune effects of this intervention. Notably, a subpopulation of monocytes was only present in the severe stage while other distinct subpopulations were present at recovery and healthy stages. Gene expression of *TNF, IL10, CCL3*, and *IL6* were significantly higher in the severe stage-specific monocytes, suggesting that a specific monocyte subpopulation may contribute to the inflammatory storm seen in patients with severe COVID-19. Additionally, interaction of IL-6/IL-6R ligand/receptor pairs were attenuated by tocilizumab treatment. Following treatment with tocilizumab, expression of genes involved in the acute inflammatory response and leukocyte chemotaxis were significantly decreased. B cells and effector CD8^+^ T cells and proliferative CD8^+^ T cells were significantly increased compared to controls, suggesting that anti-virus humoral and cell-mediated immune responses remained intact despite tocilizumab therapy. There was no difference in CD4^+^ T cells, naïve CD8^+^ T cells and B cells in patients vs. healthy controls (20).

IL-6 level was not available immediately following tocilizumab administration in our patient due to laboratory error, however, our patient's IL-6 level was found to be significantly elevated ~2 weeks after tocilizumab administration despite clinical improvement. This trend is consistent with what has been previously reported following tocilizumab treatment in patients with rheumatoid arthritis and Castleman disease. In these patients, significant rise in IL-6 level following tocilizumab treatment has been observed. IL-6 level peaked at day 14 after therapy remained at stable elevation until day 42 (21). This phenomenon has been suggested to be due to binding of tocilizumab to IL-6R leading to delayed clearance and accumulation of IL-6 in the blood (21, 22). That this elevation persists at a stable level weeks after tocilizumab administration has been suggested to be due to continued IL-6 production at approximately the same rate as direct IL-6 degredation (21).

The totality of immune effects of SARS-CoV-2 infection and tocilizumab therapy remain to be fully understood, as does the impact of treatment with tocilizumab on the clinical course in patients with AA. It is known that IL-6 impacts the balance of Th17 and Treg cells by promoting proliferation of the former and inhibiting the later (14) and that imbalance in Th17/Treg cells is an important feature in the development of AA (23). Additionally, elevation in IL-6 has been observed in children with AA compared to healthy controls (193.48 vs. 4.58 pg/mL, *p* < 0.001). The degree of IL-6 elevation was associated with severity of disease and reduction in IL-6 level was associated with response to immunosuppressive therapy (IST) (24). Others have found that children with higher baseline IL-6 elevation are more likely to respond to IST (211.89 vs. 18.09 pg/mL, *p* = 0.005) and that an IL-6 level of at least 36.8 pg/mL had 81% sensitivity to distinguish responders from non-responders (25). There are currently no published data regarding use of tocilizumab for treatment of AA, although data on the role of IL-6 in dysregulation of Th17/Treg balance and the role of this imbalance in the development of AA suggests that tocilizumab may play a role. It remains to be seen whether our patient will benefit in his blood cell counts following tocilizumab therapy.

Being a single patient report is a major limitation. However, we felt that it's prudent to report the first use of tocilizumab to treat a cytokine storm induced severe SARSCoV-2 in a patient with underlying immunodeficiency. The findings are consistent with what has been reported in immune competent patients. The rapid clinical improvement and decline of all inflammatory markers suggests that tocilizumab was effective in reversing COVID-19-associated cytokine storm.

## Patient Perspective

“I started feeling tired and had no energy even to walk short distances. I had a fever and minor cough. I went to the Emergency Room [sic] at Jefferson [sic], and I tested positive [for SARS-CoV-2] and they admitted me. The first couple of weeks (I was in the hospital for a total of 4 weeks), I slept most of the time. I was either on my back in bed or in a chair. After I started to get better, I was more awake and I became depressed and bored. I looked forward to my daily [physical therapy] visits. It is certainly difficult not having visitors. The doctors and nurses were excellent. The doctors communicated with my wife every day. I simply cannot say enough about the care I received. When released from the hospital, I came directly home and have been receiving nursing care and [physical therapy].

My case is complicated by the fact that prior to my hospitalization I required transfusions for aplastic anemia. The medical staff worked diligently to have a procedure for transfusing patients with [COVID-19] as outpatients. They were able to have a procedure set up of which I was the first. The care and attention I received at the infusion center was well-organized and excellent. My improvement is progressing and I am getting better every day.”

## Conclusion

Until now, treatment of severe COVID-19 with tocilizumab has been tried in patients without pre-existing immune dysfunction. We have reported the first use of this treatment strategy in a patient with underlying immunodeficiency. It is notable that both the symptoms and signs of severe COVID-19 and the result of therapy with tocilizumab is similar to prior reports in patients with presumably normal baseline immune function. More work is desperately needed to better understand the immunological impacts of severe SARS-CoV-2 infection and the risks and benefits of tocilizumab treatment.

## Data Availability Statement

The original contributions presented in the study are included in the article/supplementary material, further inquiries can be directed to the corresponding author/s.

## Ethics Statement

Ethical review and approval was not required for the study on human participants in accordance with the local legislation and institutional requirements. The patients/participants provided their written informed consent to participate in this study. Written informed consent was obtained from the individual(s) for the publication of any potentially identifiable images or data included in this article.

## Author Contributions

GK and ZF wrote the manuscript. LW, JF-O'H, UG, and AB reviewed and edited the manuscript. All authors contributed to the article and approved the submitted version.

## Conflict of Interest

The authors declare that the research was conducted in the absence of any commercial or financial relationships that could be construed as a potential conflict of interest.

## References

[1] Huang C Wang Y Li X Ren L Zhao J Hu Y. Clinical features of patients infected with (2019) novel coronavirus in Wuhan, China. Lancet. (2020) 395:497–506. 10.1016/S0140-6736(20)30183-5 31986264 PMC7159299

[2] Chen X Zhao B Qu Y Chen Y Xiong J Feng Y Detectable serum severe acute respiratory syndrome coronavirus 2 viral load (RNAemia) is closely correlated with drastically elevated interleukin 6 level in critically Ill patients with coronavirus disease 2019. Clin Infect Dis. (2020). 10.1093/cid/ciaa449. [Epub ahead of print]. PMC7184354 32301997

[3] Ruan Q Yang K Wang W Jiang L Song J Clinical predictors of mortality due to COVID-19 based on an analysis of data of 150 patients from Wuhan, China. Intensive Care Med. (2020) 46:846–8. 10.1007/s00134-020-06028-z 32125452 PMC7080116

[4] Mehta P McAuley DF Brown M Sanchez E Tattersall RS Manson JJ. COVID-19: consider cytokine storm syndromes and immunosuppression. Lancet. (2020) 395:1033–4. 10.1016/S0140-6736(20)30628-0 32192578 PMC7270045

[5] Zhang S Li L Shen A Chen Y Qi Z. Rational use of tocilizumab in the treatment of novel coronavirus pneumonia. Clin Drug Investig. (2020) 40:511–8. 10.1007/s40261-020-00917-3 32337664 PMC7183818

[6] Luo P Liu Y Qiu L Liu X Liu D Li J. Tocilizumab treatment in COVID-19: a single center experience. J Med Virol. (2020) 92:814–8. 10.1002/jmv.25801 32253759 PMC7262125

[7] Michot JM Albiges L Chaput N Saada V Pommeret F Griscelli F. Tocilizumab, an anti-IL6 receptor antibody, to treat Covid-19-related respiratory failure: a case report. Ann Oncol. (2020) 31:961–4. 10.1016/j.annonc.2020.03.300 32247642 PMC7136869

[8] Di Giambenedetto S Ciccullo A Borghetti A Gambassi G Landi F Visconti E. Off-label use of tocilizumab in patients with SARS-CoV-2 infection. J Med Virol. (2020). 10.1002/jmv.25897. [Epub ahead of print]. 32297987 PMC7262080

[9] Xu X Han M Li T Sun W Wang D Fu B. Effective treatment of severe COVID-19 patients with tocilizumab. Proc Natl Acad Sci USA. (2020) 117:10970–5. 10.1073/pnas.2005615117 32350134 PMC7245089

[10] Wang D Hu B Hu C Zhu F Liu X Zhang J. Clinical characteristics of 138 hospitalized patients with 2019 novel coronavirus-infected pneumonia in Wuhan, China. JAMA. (2020) 323:1061–9. 10.1001/jama.2020.1585 32031570 PMC7042881

[11] Liu J Li S Liu J Liang B Wang X Wang H. Longitudinal characteristics of lymphocyte responses and cytokine profiles in the peripheral blood of SARS-CoV-2 infected patients. EBioMedicine. (2020) 55:102763. 10.1016/j.ebiom.2020.102763 32361250 PMC7165294

[12] Zhou F Yu T Du R Fan G Liu Y Liu Z Clinical course and risk factors for mortality of adult inpatients with COVID-19 in Wuhan, China: a retrospective cohort study. Lancet. (2020) 395:1054–62. 10.1016/S0140-6736(20)30566-3 32171076 PMC7270627

[13] Teachey DT Lacey SF Shaw PA Melenhorst JJ Maude SL Frey N. Identification of predictive biomarkers for cytokine release syndrome after chimeric antigen receptor T-cell therapy for acute lymphoblastic leukemia. Cancer Discov. (2016) 6:664–79. 10.1158/2159-8290.CD-16-0040 27076371 PMC5448406

[14] Tanaka T Narazaki M Kishimoto T IL-6 in inflammation, immunity, and disease. Cold Spring Harb Perspect Biol. (2014) 6:a016295 10.1101/cshperspect.a016295 25190079 PMC4176007

[15] Madkaikar M Ghosh K Gupta M Swaminathan S Mohanty D. *Ex vivo* expansion of umbilical cord blood stem cells using different combinations of cytokines and stromal cells. Acta Haematol. (2007) 118:153–9. 10.1159/000108630 17890847

[16] Tanaka T Narazaki M Ogata A Kishimoto T. A new era for the treatment of inflammatory autoimmune diseases by interleukin-6 blockade strategy. Semin Immunol. (2014) 26:88–96. 10.1016/j.smim.2014.01.009 24594001

[17] Grupp SA Laetsch TW Buechner J Bittencourt H Maude SL Verneris MR Analysis of a global registration trial of the efficacy and safety of CTL019 in pediatric and young adults with relapsed/refractory acute lymphoblastic leukemia (ALL). Blood. (2016) 128:221 10.1182/blood.V128.22.221.221

[18] Roumier M Paule R Groh M Vallee A Ackermann F Interleukin-6 blockade for severe COVID-19. medRxiv [Preprint]. (2020). 10.1101/2020.04.20.20061861

[19] Li G Fan Y Lai Y Han T Li Z Zhou P Coronavirus infections and immune responses. J Med Virol. (2020) 92:424–32. 10.1002/jmv.25685 31981224 PMC7166547

[20] Guo C Li B Ma H Wang X Cai P Yu Q. Single-cell analysis of two severe COVID-19 patients reveals a monocyte-associated and tocilizumab-responding cytokine storm. Nat Commun. (2020) 11:3924. 10.1038/s41467-020-17834-w 32764665 PMC7413381

[21] Nishimoto N Terao K Mima T Nakahara H Takagi N Kakehi T. Mechanisms and pathologic significances in increase in serum interleukin-6 (IL-6) and soluble IL-6 receptor after administration of an anti–IL-6 receptor antibody, tocilizumab, in patients with rheumatoid arthritis and Castleman disease. Blood. (2008) 112:3959–64. 10.1182/blood-2008-05-155846 18784373

[22] Uchiyama Y Yoshida H Koike N Hayakawa N Sugita A Nishimura T Anti-IL-6 receptor antibody increases blood IL-6 level via the blockade of IL-6 clearance, but not via the induction of IL-6 production. Int Immunopharmacol. (2008) 8:1595–601. 10.1016/j.intimp.2008.07.002 18664393

[23] Zeng Y Katsanis E. The complex pathophysiology of acquired aplastic anaemia. Clin Exp Immunol. (2015) 180:361–70. 10.1111/cei.12605 25683099 PMC4449765

[24] Gupta V Kumar S Sonowal R Singh SK. Interleukin-6 and interleukin-8 levels correlate with the severity of aplastic anemia in children. J Pediatr Hematol Oncol. (2017) 39:214–6. 10.1097/MPH.0000000000000724 28060106

[25] Lu S Qiao X Xie X. Elevated serum interleukin-6 predicts favorable response to immunosuppressive therapy in children with aplastic anemia. J Pediatr Hematol Oncol. (2017) 39:614–7. 10.1097/MPH.0000000000000942 29068868

